# A “U-shaped" Curve: Appreciating How Primary Care Residency Intention Relates to the Cost of Board Preparation and Examination

**DOI:** 10.7759/cureus.5613

**Published:** 2019-09-10

**Authors:** Philip A Bucur, Vikrant Bhatnagar, Sebastian R Diaz

**Affiliations:** 1 Miscellaneous, Ohio University Heritage College of Osteopathic Medicine, Athens, USA; 2 Family Medicine, Ohio University Heritage College of Osteopathic Medicine, Athens, USA

**Keywords:** primary care, medical education debt, board examination, board preparation, costs, primary care physician shortage, residency intention, osteopathic medical students, medical student career choice, board examination and preparation costs

## Abstract

Introduction: The shortage of primary care physicians in the United States has warranted an investigation into how medical education debt and other factors influence medical students’ interests in primary care (PC) residencies. However, sparse research has studied how the cost of board preparation and examination relates to career choice. The objective of this study was to determine if there is an association between the cost of preparing and sitting for board examinations and the intention to enter a PC residency for osteopathic medical students.

Methods: We postulated that students who incurred higher financial costs from preparing and sitting for board examinations would be more likely to be interested in non-primary care (NPC) residencies. Using a non-experimental survey design, this study asked respondents to evaluate the following: “I plan to enter a Primary Care Residency (Family Medicine OR General Internal Medicine OR Pediatrics)” using a Likert scale. Respondents were also asked to select which board examination(s) and pertinent resource(s) they had purchased. Total costs were calculated per student.

Results: A total of 25,852 osteopathic medical students received the survey, of which 1,280 students responded to and completed it, yielding a 4.95% response rate. The distribution of respondents’ intentions to pursue a primary care residency and costs spent yielded a “U” shaped curve. Respondents who Strongly Agreed and Strongly Disagreed to the statement “I plan to enter a Primary Care Residency” spent $5,744 and $5,070 on board-preparation and examination, respectively. No statistically significant differences were found between the cost of preparing and sitting for board examinations and the intention to enter primary care residencies when individuals were grouped by year in school and gender.

Conclusions: Because competitive NPC specialties have relatively higher salaries, we suspected that students who intended to pursue these specialties would have had higher financial costs from board examination and preparation compared to students who intended to pursue PC residencies such as family medicine. Our findings further illustrate these specific educational costs do not correlate with students’ stated intentions to enter primary care residencies. As efforts continue to determine a solution for the primary care physician shortage, it becomes clearer that the focus must also encompass non-financial influences that shape career choice.

## Introduction

The shortage of primary care physicians (PCPs) has hindered healthcare delivery. States with higher PCP to population ratios are found to have lower all-cause mortality rates and improved health outcomes [1]. With the number of PCPs expected to grow 7% by 2025, there will still be a shortage of 44,000 PCPs [2]. Patient dissatisfaction, growing workload demand, and physician reimbursements have exacerbated this shortage [3]. While salaries for family physicians have increased to an average of $223,000, the salary gap between family physicians and specialists is approximately $106,000 [4]. These are some issues that might deter U.S. medical graduates from entering primary care fields. Family medicine residencies saw a 50% decrease in medical school graduates entering the field between 1997 and 2005 [3].

Osteopathic institutions, which graduated 6,015 medical students in the 2016-2017 academic year, or 32% of all US medical graduates, have focused their efforts in increasing the number of PCPs [5,6]. For example, one-third of osteopathic graduates in 2013 had intended to pursue primary care careers, while another one-third had intended to practice in underserved areas [7]. To leverage this trend among osteopathic institutions toward addressing the PCP shortage, it is imperative to determine what draws medical school graduates toward non-primary care (NPC) residencies and away from primary care (PC) residencies.

Medical student debt has been found to have little significance in determining a medical student’s career choice. Viewing medical students as a whole, anticipated medical student debt has been found to have little to no association with the intention to pursue a primary care residency [8-10]. For example, the length of training has been revealed to be a more important factor than financial compensation for students pursuing PC careers [8]. Students with any level of debt were twice as likely to pursue a PC career compared to students without a debt [8]. Moreover, the interest in entering an internal medicine residency, a field within primary care, is low regardless of educational debt level accumulated [11].

In combination, however, anticipated income and debt may influence residency choice. For instance, fourth-year medical students who switched from pursuing a PC to NPC residency estimated having higher debt, while also anticipated a higher income compared to students pursuing PC residencies [12]. Whether a student attends a private or public medical school may affect educational debt and, thus, PC residency intention. Debt levels above $100,000 deter public medical school graduates from pursuing PC residencies [13]. Meanwhile, at private medical schools, debt levels above $100,000 did not correlate with PC and NPC career choice [13]. Although student loan indebtedness had little overall influence, PC residency career choices have been found to be more influenced by student loan debt relative to surgical residency career choices [14].

To become licensed physicians, osteopathic medical students are required to take the three-level Comprehensive Osteopathic Medical Licensing Examination of the United States (COMLEX-USA) board examinations, while allopathic students must take the three-step United States Medical Licensing Examination (USMLE) boards [15,16]. Many osteopathic medical students elect to take the USMLE Step 1 in addition to their required COMLEX-USA Level 1 exam to increase their competitiveness for residencies accredited by the Accreditation Council for Graduate Medical Education (ACGME) [17].

Although a passing score is required for licensing, board exam performance is a significant metric used by program directors to determine who to interview during the residency selection process [18]. Across all specialties, programs directors rank USMLE Step 1 scores as the 2^nd^ highest criteria for applicant selection [19]. Specifically, family medicine program directors are increasingly valuing objective application criteria such as USMLE Step 2 Clinical Knowledge scores, with many ranking it as the second most important criteria [19]. Concerned about board performance, students augment their learning by purchasing review materials such *as First Aid for the USMLE Step 1* and *Pathoma* from third-party for-profit vendors [18]. 

Purchasing a wide variety of these resources over the course of medical school can accumulate significant costs, directly adding to medical school debt. Financial costs incurred while preparing and sitting for board examinations have been found to represent 2.94% of average osteopathic medical student debt for the class of 2018 [20]. The objective of this study was to determine if there is an association between the cost of taking and preparing for board examinations and the intention to enter a PC residency for osteopathic medical students.

## Materials and methods

We hypothesized that students with higher financial costs from preparing and sitting for board examinations were more likely to pursue NPC residencies compared to PC residencies. This Institutional Review Board (IRB)-approved study used a non-experimental survey design to assess osteopathic medical students’ intentions to enter primary care residencies in relation to the financial costs they had incurred or expect to incur from board preparation and examination. To gain a comprehensive representation of the diverse backgrounds of osteopathic medical students, a nationwide survey was administered using Qualtrics software (Qualtrics Inc, Provo, USA). Participant anonymity was ensured through software settings that prevented the collection of Internet Protocol (IP) addresses.

The Student Government Association (SGA) President at Ohio University Heritage College of Osteopathic Medicine contacted SGA Presidents at 38 osteopathic medical schools via email to distribute the survey link to their respective medical school student bodies. The survey questions showed key words, highlighted in bold text, to increase the clarity of each question. The survey was available for two weeks to allow respondents sufficient time to complete it.

The online survey consisted of 12 questions, of which the first to fifth survey questions focused on demographic information (see Appendices). Using a fill in the circle option, respondents were asked to indicate in which of the four regions (Northeast, South, West and Midwest) of the United States their medical school was located, gender (male, female, or other), their race, age of matriculation (under age 25, 25 - 30, or over age 30), and current academic year.

The sixth survey question asked participants to select which of the following board examinations they took or plan to take, with COMLEX exams being mandatory for osteopathic medical students and USMLE exams generally being optional: 1) COMLEX-USA Level 1; 2) USMLE Step 1; 3) COMLEX-USA Level 2 Cognitive Evaluation (CE); 4) USMLE Step 2 Clinical Knowledge (CK); 5) COMLEX-USA Level 2 Performance Evaluation (PE), and; 6) USMLE Step 2 Clinical Skills (CS). Four options were provided for each exam: 1) never taken but plan to take; 2) never taken and do not plan to take; 3) taken once, and; 4) taken more than once.

The seventh survey question asked participants to select the listed options of resources that they had purchased or planned to purchase in preparation for COMLEX-USA Level 1/USMLE Step 1. The resources were listed in alphabetical order so that a respondent could navigate and find their resources more easily. An option to fill-in “other” resources that were not listed was also provided on the survey. Due to an oversight error, the resource First Aid for the USMLE Step 1 was not included in the list of options.

After respondents selected their purchased review materials, the eighth survey question asked respondents to indicate the number of practice exams they had purchased or planned to purchase in preparation for COMLEX-USA Level 1/USMLE Step 1. The options included a scale from zero exams to six exams (the maximum available for purchase) for National Board of Medical Examiners (NBME) Step 1 practice exams and zero exams to three exams (the maximum available for purchase) for National Board of Osteopathic Medical Examiners (NBOME) Comprehensive Osteopathic Medical Self-Assessment Examination (COMSAE) Phase 1 practice exams. 

The ninth survey question asked respondents to select the listed options of resources that they had purchased or planned to purchase in preparation for COMLEX-USA Level 2 CE/USMLE Step 2 CK. The resources were also listed in alphabetical order so that a respondent could navigate and find their resources more easily. Again, an option to fill-in "other" resources that were not listed was provided on the survey.

The tenth survey question asked respondents to select the alphabetically listed options of resources that they had purchased or planned to purchase to prepare for COMLEX-USA Level 2 PE/USMLE Step 2 CS. Respondents had the option to fill-in "other" resources that were not provided as a list of options as well. 

The eleventh survey question asked respondents to indicate the number of practice exams they had purchased or planned to purchase in preparation for COMLEX-USA Level 2 CE/USMLE Step 2 CK, and COMLEX-USA Level 2 PE/USMLE Step 2 CS. The options included a scale from zero exams to three exams (the maximum available for purchase) for NBME Step 2 CK practice exams, zero exams to two exams (the maximum available for purchase) for NBME Step 2 CS practice exams, and zero exams to one exam (the maximum available for purchase) for the NBOME Level 2 CE practice exam.

Lastly, respondents’ attitudes toward the statement - “I plan to enter a Primary Care Residency (Family Medicine OR General Internal Medicine OR Pediatrics)” was assessed using an ordinal Likert scale (1 - Strongly Disagree, 2 - Disagree, 3 - Neutral, 4 - Agree, and 5 - Strongly Agree) for the twelfth survey question. 

The price for each resource was attained by accessing the resources’ respective websites and finding the “list price” of a brand-new copy or version of that resource. If a price was not found, the current Amazon.com “list price” was utilized. Responses were analyzed using Statistical Package for the Social Sciences (SPSS) statistical software version 24.0 (SPSS Inc, Chicago, USA). Analysis of Variance (ANOVA) was employed to compare the means of multiple categories. Post-hoc comparisons of levels of categorical independent variables employed the Scheffé test. Since levels of the independent variable are ordinal (i.e., comparing outcomes amongst those who Strongly Disagreed, Disagreed, were Neutral, Agreed and Strongly Agreed to the statement), the Scheffé test was used given its more conservative statistical power as compared to other post-hoc comparison statistics. Inferential statistical tests utilized the conventional p=0.05 cutoff.

## Results

**Figure 1:**
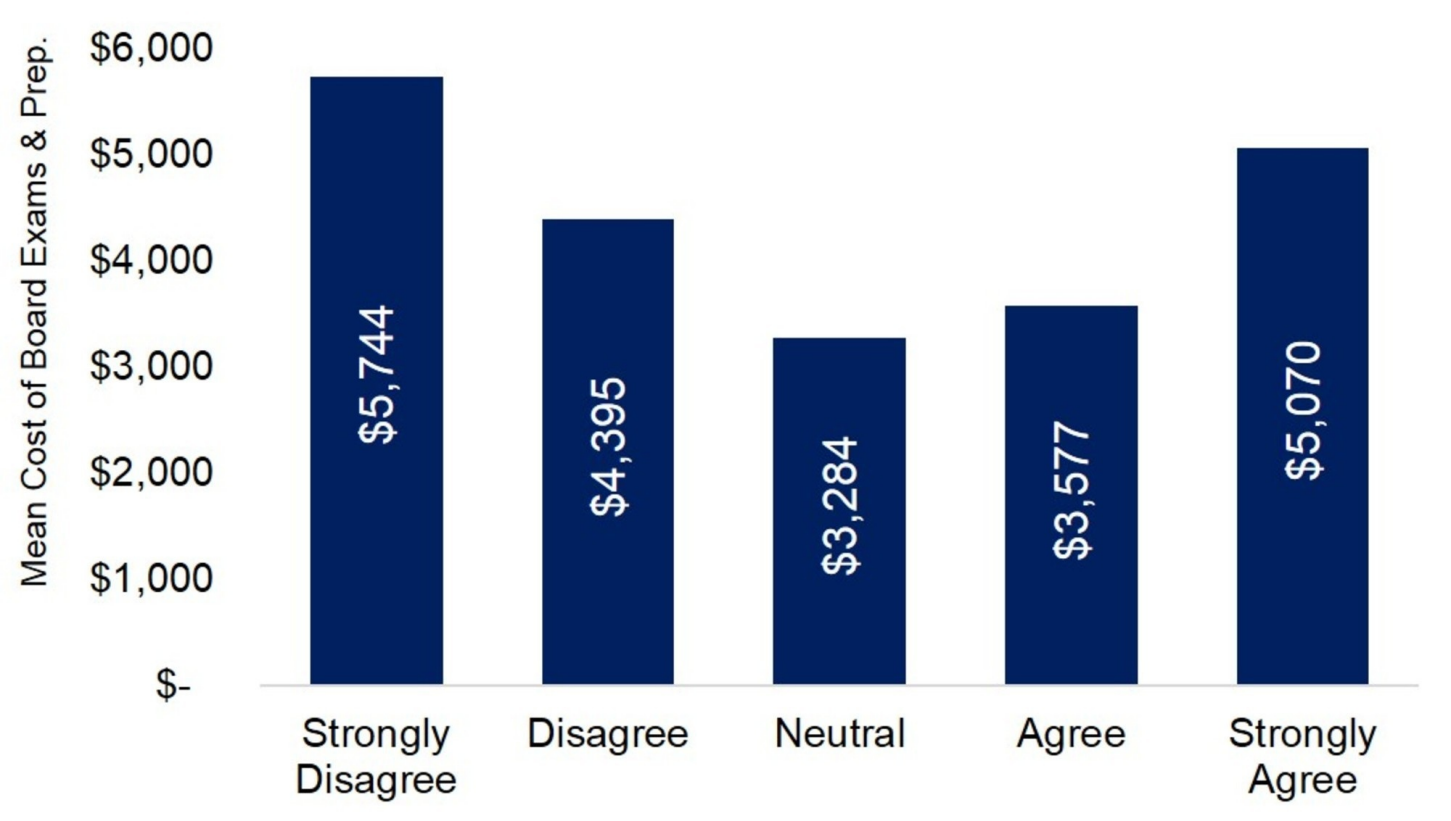
The Mean Cost of Board Examination and Preparation Together when Evaluating the Statement: “I Plan to Enter a Primary Care Residency.” A unique “U” shaped distribution develops when the mean cost of board preparation and examination is plotted against the five Likert options. Post-hoc comparisons revealed no statistically significant difference between respondents who Strongly Disagreed and Strongly Agreed (p=0.168).

Of the (n=25,852) osteopathic medical students from 38 osteopathic medical schools who received the survey link, 1,280 osteopathic medical students responded, providing a 4.95% response rate. In our sample of respondents, African American students represented 2.8%, Asian students represented 14.2%, and White students represented 77.9%. When the mean cost of board preparation and examination is plotted against the five Likert options (Strongly Disagree, Disagree, Neutral, Agree and Strongly Agree) for the statement “I plan to enter a Primary Care Residency”, a “U” shaped distribution is observed (Figure 1). Respondents who Strongly Disagreed and Strongly Agreed with the statement spent $5,744 and $5,070, respectively, while individuals who Disagreed and Agreed spent $4,395 and $3,577, respectively, and Neutral individuals accrued $3,284 (Figure 1). ANOVA detected a statistically significant difference among Likert scale response categories (F=24.24; df=4; p<0.001). Post-hoc comparisons revealed statistically significant differences between those who Strongly Disagreed and those who Disagreed, were Neutral, and Agreed (all comparisons at p<0.001). No statistically significant difference was detected between respondents who Strongly Disagreed and Strongly Agreed (p=0.168).

**Figure 2:**
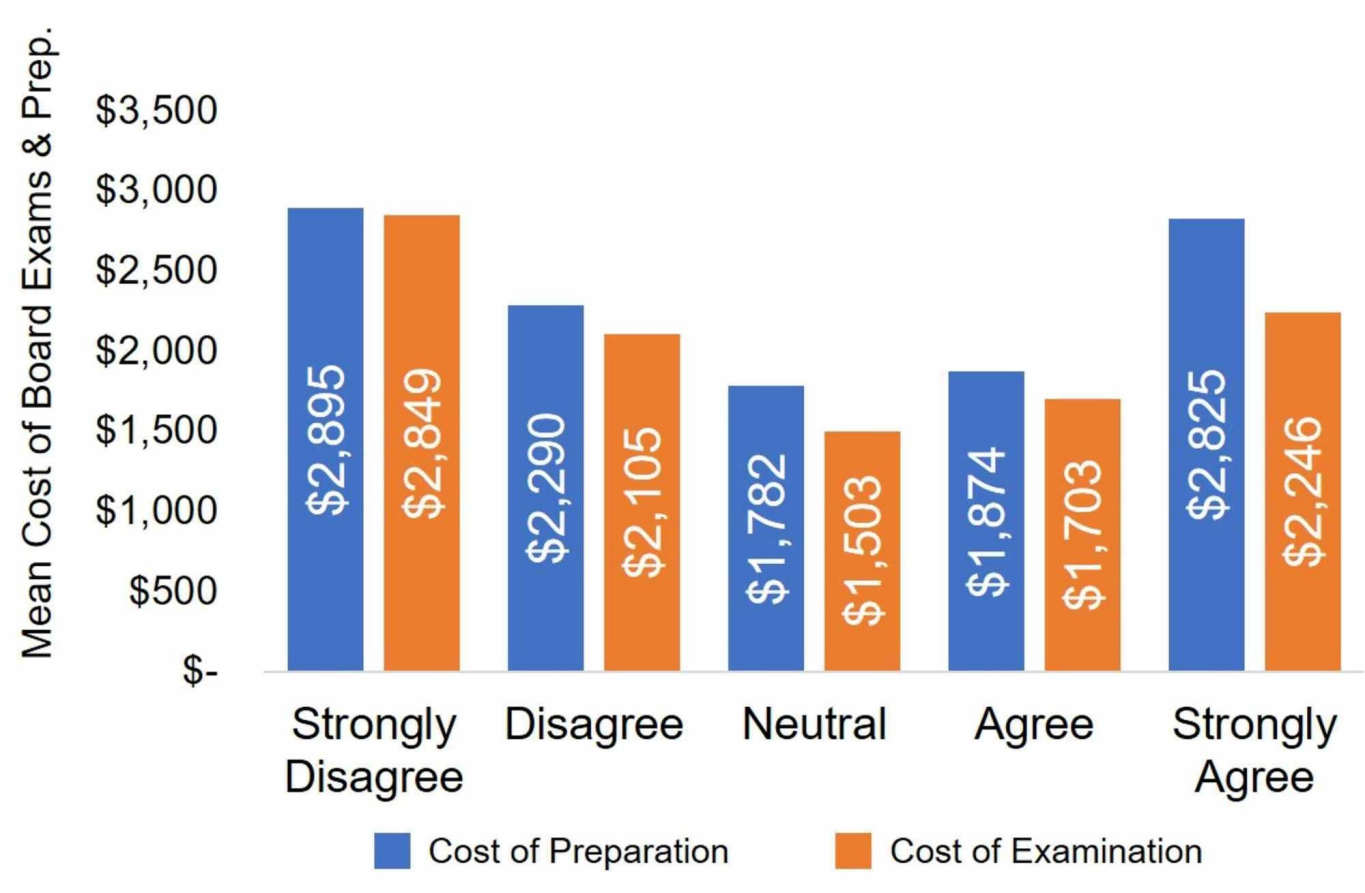
The Mean Cost of Board Examination and Preparation Separately when Evaluating the Statement: “I Plan to Enter a Primary Care Residency.” The “U” shaped curve persists when bifurcating the costs into preparation and examination. For preparation costs, no statistically significant difference was found between those who Strongly Disagreed and Strongly Agreed (p=0.998), as well as those who Strongly Disagreed and Disagreed (p=0.113). However, when isolating examination cost, post-hoc comparisons revealed statistically significant differences between those who Strongly Disagreed and those who Disagreed, were Neutral, Agreed, and Strongly Agreed (p<0.001).

After bifurcating the total cost into (1) cost of board preparation, and (2) cost of board examinations, this “U” shaped pattern persists (Figure 2). When focusing on the cost of board preparation, respondents who Strongly Disagreed and Strongly Agreed with the statement spent $2,895 and $2,825, while individuals who Disagreed and Agreed spent $2,290 and $1,874, and Neutral individuals spent $1,782 (Figure 2). ANOVA detected a statistically significant difference among Likert scale response categories (F=11.62; df=4; p<0.001). Post-hoc comparisons revealed statistically significant differences between those who Strongly Disagreed and those who were Neutral and Agreed (p<0.001). No statistically significant difference was detected between those who Strongly Disagreed and Strongly Agreed with the statement (p=0.998), as well as those who Strongly Disagreed and Disagreed with the statement (p=0.113).

When isolating the cost of board examinations, respondents who Strongly Disagreed and Strongly Agreed with the statement spent $2,849 and $2,246, respectively, while individuals who Disagreed and Agreed spent $2,105 and $1,703, respectively, with Neutral individuals accruing $1,503 (Figure 2). ANOVA detected a statistically significant difference among Likert scale response categories (F=42.90; df=4; p<0.001). Post-hoc comparisons revealed statistically significant differences between those who Strongly Disagreed and those who Disagreed, were Neutral, Agreed, and Strongly Agreed (p<0.001).

**Figure 3:**
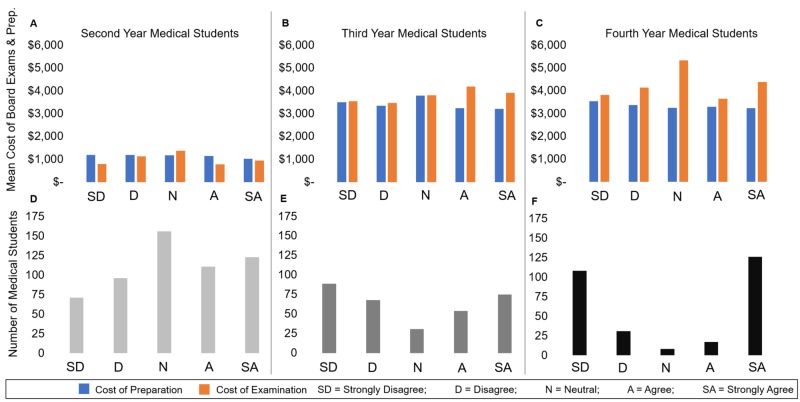
The Cost of Board Examination and Preparation Separately and Number of Respondents when Evaluating the Statement “I Plan to Enter a Primary Care Residency” by Year in School The costs of both preparation and examination increase as a respondent progresses through medical school. (A) the mean costs of board examination and preparation incurred by second-year medical students when plotted against the five Likert options (Strongly Disagree, Disagree, Neutral, Agree, Strongly Agree). (B, C) the mean costs of board examination and preparation incurred by the third- and fourth-year medical students, respectively, when plotted against the same Likert options.  When the number of respondents is plotted against the five Likert options, more second-year students were found to be neutral (D), with third-year students exhibiting the early signs of a “U” shaped curve (E), and fourth-year students showcasing the full “U” shape (F).

The costs of both preparation and examination increase as a respondent progresses through medical school (Figure 3). Board examinations are most commonly taken at the end of Year-2 and Year-3 of medical school, suggesting that third and fourth-year medical students should accrue similar amounts (Figure 3B, 3C). Interestingly, when the number of respondents is plotted against the five Likert options, more second-year students were found to be neutral, with third-year students exhibiting the early signs of a “U” shaped curve, and fourth-year students showcasing the full “U” shape (Figures 3D-3F).

When examining the change in the number of students who intend to enter PC and NPC residencies over the medical school continuum, 26% more third-year medical students Strongly Disagreed to the statement compared to second-years, while 23% more fourth-year medical students Strongly Disagreed compared to third years. Comparatively, the number of neutral respondents decreased by 80% from the second to the third year, and by 74% from the third year to the fourth year.

**Figure 4:**
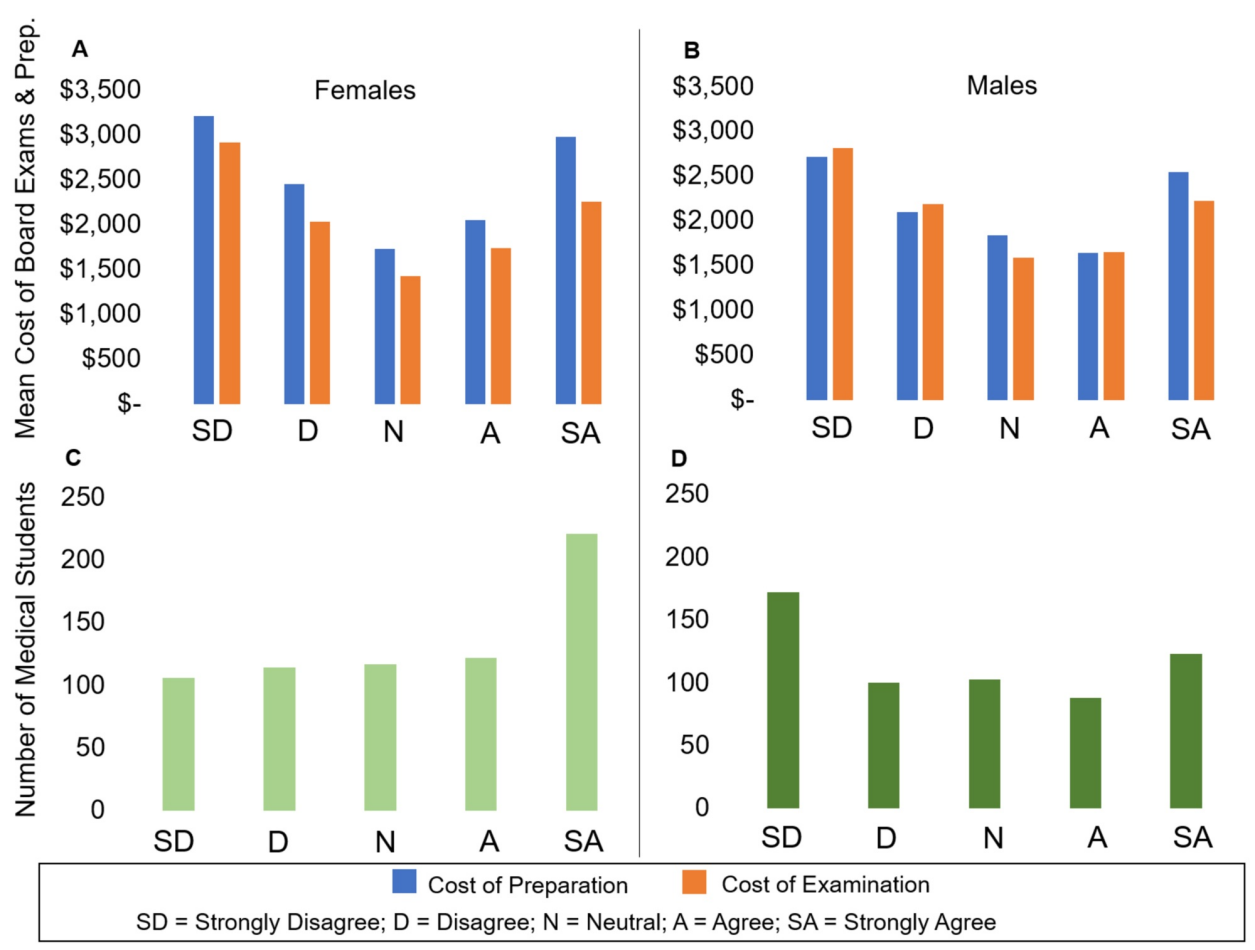
The Cost of Board Examination and Preparation Separately and Number of Respondents when Evaluating the Statement “I Plan to Enter a Primary Care Residency” by Gender Respondents evaluating the statement were grouped by gender and the costs of preparation and examination were calculated separately for each gender. The number of respondents by gender evaluating the statement was also determined. (A, B) Although the “U” shape distribution is again visualized when the costs of both preparation and examination are plotted by gender, the distribution of respondents by Likert responses is more polarized amongst the genders. (C) 33% of females chose Strongly Agree to the aforementioned statement while (D) 30% of males selected Strongly Disagree when asked about their intent to pursue primary care.

The “U” shaped curve is again visualized when the costs of both preparation and examination are plotted by gender (Figures 4A, 4B). However, the distribution of respondents by Likert responses is more polarized among the genders, with 33% of females choosing Strongly Agree and 30% of males selecting Strongly Disagree when asked about their intent to pursue primary care (Figures 4C, 4D). Moreover, 29% more female respondents Strongly Agreed to the statement compared to male respondents.

## Discussion

Medical students’ intentions to enter PC residencies in relation to educational debt has been extensively studied. However, this study uniquely sought to determine if the costs of board preparation and examination, a direct factor to overall educational debt, are associated with osteopathic medical student intention to enter PC residencies [20]. The findings from this study can be summarized by the “U” shaped curve that we discuss.

No statistically significant difference was found between the mean amount spent on board preparation and examination of those who Strongly Agreed and Strongly Disagreed with the statement “I plan to enter a Primary Care Residency (Family Medicine OR General Internal Medicine OR Pediatrics).” This finding provides evidence that, like overall debt, board preparation spending is not reflective of residency choice. It indicates that students who desire to enter a PC residency will spend as much on board preparation and examination compared to students who want to enter an NPC residency. Furthermore, this finding is supported by evidence that individuals inclined to purse a PC residency are potentially more influenced by non-financial factors: growing up in a rural area, having interest in serving the underserved, and attending public, rural medical schools [21]. The scope and diverse experiences in clinical practice involved with diverse patient problems are also associated with PC residency interest [22].

Since students who Strongly Agreed and Strongly Disagreed spent nearly the same amount for preparing and taking board exams, further exploration is warranted to understand these students’ viewpoints on board examination performance. As of the 2018 Main Residency Match, family medicine had the lowest mean COMLEX-USA Level 1 score of matched osteopathic graduates at 506 (USMLE Step 1 = 217) [23]. As previously discussed, students augment their curriculum and board studying by purchasing review materials to optimize their performance on exams [18]. Based on our results, one may infer that students interested in PC residencies are as concerned about board performance compared to those interested in NPC residencies, using dollars spent as a proxy to measure concern for performance. 

The “U” shaped curve re-occurs when evaluating the intention to enter PC residencies while progressing through the medical school curriculum. This highlights the importance of clinical education during the third and fourth year, with most medical students changing their desired specialty by fourth-year [24]. As previously stated, 26% more third-year medical students Strongly Disagreed to the statement compared to second years while 23% more fourth-year medical students Strongly Disagreed compared to third years. Comparatively, the number of neutral respondents decreased by 80% from the second to third year, and by 74% from third year to fourth year. This trend has been validated by another study which found that a majority of students entering medical school undecided in career choice elected to enter an NPC residency by their fourth year [24]. Moreover, a significant number of students see their third year of medical school as the time they decide on career specialty [25]. With no statistically significant difference between the costs incurred from preparing and sitting for board examinations of those who Strongly Agreed and Strongly Disagreed to the statement "I Plan to Enter a Primary Care Residency," our study further supports the theory that financial costs during undergraduate medical training are not a significant factor in career choice.

When viewing the intent to enter PC residencies by gender, differences were identified; 29% more female respondents Strongly Agreed to the statement than male respondents. This follows the current trends of female physicians practicing in primary care settings. As of 2015, 58% of family medicine residents and 75% of pediatric residents are female, while 46% of internal medicine residents are female [26]. Additionally, PC residency interest has been found to be higher among women compared to men [27]. Interestingly, the “U” shaped curve was present for male and female respondents in relation to the mean amount spent on board exam preparation and intention to enter PC residencies.

Limitations

Several limitations inform the generalizability of this study.First, due to an oversight error, 'First Aid for the USMLE Step 1' was not included in the survey. In a previous study, the average cost of board preparation and examination for osteopathic medical students was found to be $7,499 [20]. This resource has a list price of $55, 0.73% of the average cost calculated in the previous study [20]. As a result, we believe this omission does not significantly alter the results of this study. Moreover, some study participants may have purchased resources that were previously used, likely resulting in a lower purchase price. Although this may decrease the average cost a student may incur, we suspect that this would not have a drastic effect on the curve we witness. However, further studies may investigate the number of resources a student may purchase to obtain a competitive score on board examinations in relation to intended residency specialty.

Second, the sample size of respondents who were fourth-year medical students was lower compared to non-fourth-years. The small sample size can be attributed to the timing of the survey distribution when a majority of fourth-year medical students were completing interviews for post-graduate medical training. Because of this smaller sample size, the influence of fourth-year students’ perceptions is underrepresented in our statistical models. Representation of these students' perceptions is important because it has been found that a majority of students who enter medical school, undecided on career choice, elect to apply for NPC residencies by their fourth year [25].

Moreover, while (n=1,280) responses represent only 4.95% of osteopathic medical students nationwide, this low response rate may be attributed to survey fatigue experienced by medical students who receive numerous requests throughout the year to complete surveys. In contrast to others, no monetary incentive was provided for students to complete this survey, potentially lowering the response rate. However, we believe that the low response rate did not affect our findings because our sample comprises students across the United States and we obtained a sample size large enough to appreciate the “U” shaped curve. Future studies should be employed at a time when many students are not potentially studying for board examinations and away during residency interviews. 

Additionally, our sample demographics did not completely reflect those enrolled in osteopathic medical schools. Of students who matriculated nationwide in 2017, 59.8% were white, 24.4% were Asian, 7.5% were Hispanic/Latino, and 3.2% were African American [28]. African American students represented 2.8% of our sample, Asian students represented 14.2% and White students represented 77.9%. Although our sample reflected the African American demographic well to that of osteopathic schools nationally, our study was not reflective of the Asian demographic and over-represented the White demographic. The difference in demographics limits the generalizability of our results to underrepresented races and/or ethnicities.

Lastly, this survey was limited to osteopathic medical students. While osteopathic medical students comprise 33% of total medical students in the United States, allopathic medical students should be included in future studies[29,30]. This will lead to a better understanding of the association between primary care residency intention and the cost of board examination and preparation for all medical students.

## Conclusions

This study sought to answer if the costs of board preparation and examination were associated with medical students’ intention to enter a primary care residency. As a factor in overall medical student debt, it was hypothesized that these additional financial costs influence medical students to apply for NPC over PC residencies. In accordance with previous literature regarding financial factors' non-influential role in career choice, this study found that there was no statistically significant difference in costs incurred for those who Strongly Agreed and Strongly Disagreed with the statement “I plan to enter a Primary Care Residency.” Thus, factors such as experience during clinical years and lifestyle may play a larger role in determining if one chooses a PC or NPC residency.

## Appendices

Appendix A: Survey

By clicking "Yes" below, you will consent to participate in this short survey. Do you consent to participate in this study?

o Yes, I consent (I may withdraw participation at any time). (1)

o No, I do not consent. (2)

Q1 In which region of the USA is your medical school located?

o Northeast (1)

o Midwest (2)

o West (3)

o South (4)

Q2 With which of the following do you primarily identify?

o Male (1)

o Female (2)

o Other (3)

Q3 With which of the following do you primarily identify?

▢ White (1)

▢ Black or African American (2)

▢ American Indian or Alaska Native (3)

▢ Asian (4)

▢ Native Hawaiian or Pacific Islander (5)

▢ Other (6)

Q4 At what age did you BEGIN Medical School?

o Under age 25 (1)

o Between ages 25 and 30 (2)

o Over age 30 (3)

Q5 In which year of medical school are you CURRENTLY enrolled?

o OMS - I (1)

o OMS - II (2)

o OMS - III (3)

o OMS - IV (4)

o Other (e.g. dual degree, etc.) (5)

Q6 Which of the following board examinations have you taken? 

COMLEX-USA Level 1 (1)

o  Never Taken, But Plan to Take (1)

o  Never Taken, And Do NOT Plan to Take (2)

o  Taken Once (3)

o  Taken More than Once (4)

USMLE Step 1 (2)

o  Never Taken, But Plan to Take (1)

o  Never Taken, And Do NOT Plan to Take (2)

o  Taken Once (3)

o  Taken More than Once (4)

COMLEX-USA Level 2 CE (3)

o  Never Taken, But Plan to Take (1)

o  Never Taken, And Do NOT Plan to Take (2)

o  Taken Once (3)

o  Taken More than Once (4)

 USMLE  Step 2 CK (4)

o  Never Taken, But Plan to Take (1)

o  Never Taken, And Do NOT Plan to Take (2)

o  Taken Once (3)

o  Taken More than Once (4)

COMLEX-USA Level 2 PE (5)

o  Never Taken, But Plan to Take (1)

o  Never Taken, And Do NOT Plan to Take (2)

o  Taken Once (3)

o  Taken More than Once (4)

USMLE  Step 2 CS (6)

o  Never Taken, But Plan to Take (1)

o  Never Taken, And Do NOT Plan to Take (2)

o  Taken Once (3)

o  Taken More than Once (4)

Q7 Please select all resources you've purchased or plan to purchase in preparation for COMLEX-USA Level 1 / USMLE Step 1 Board Examinations.

▢ N/A (1)

▢ Acid-Base, Fluids, and Electrolytes Made Ridiculously Simple (2)

▢ Anatomy - An Essential Textbook (3)

▢ Anatomy Flash Cards: Anatomy on the Go (4)

▢ Anki (5)

▢ Atlas of Anatomy - Thieme (6)

▢ Basic Immunology (7)

▢ Becker - GuideMD (8)

▢ Becker - Integrated Cases (9)

▢ Becker - Integrated Final Review (10)

▢ Becker - Live Intensive (11)

▢ Becker - Live Online (12)

▢ Becker - Live Review (13)

▢ Becker - QMD (14)

▢ Becker - USMLE Textbooks (15)

▢ Blue Histology (16)

▢ Boards and Beyond (17)

▢ BRS Behavioral Science (18)

▢ BRS Biochemistry, Molecular Biology, and Genetics (19)

▢ BRS Cell Biology and Histology (20)

▢ BRS Embryology (21)

▢ BRS Pathology (22)

▢ BRS Pharmacology (23)

▢ BRS Physiology (24)

▢ BRS Physiology Cases and Problems (25)

▢ Case Files Anatomy (26)

▢ Case Files Biochemistry (27)

▢ Case Files Microbiology (28)

▢ Case Files Neuroscience (29)

▢ Case Files Pharmacology (30)

▢ Case Studies in Immunology: A Clinical Companion (31)

▢ Clinical Anatomy Made Ridiculously Simple (32)

▢ Clinical Biochemistry Made Ridiculously Simple (33)

▢ Clinical Biostatistics and Epidemiology Made Ridiculously Simple (34)

▢ Clinical Microbiology Made Ridiculously Simple (35)

▢ Clinical Neuroanatomy Made Ridiculously Simple (36)

▢ Color Atlas of Physiology (37)

▢ COMBANK Question Bank (38)

▢ COMQUEST Question Bank (39)

▢ Cracking the Boards: USMLE Step 1 (40)

▢ Cracking the USMLE Step 1 (41)

▢ Cram Fighter (42)

▢ Crash Course: Anatomy (43)

▢ Crash Course: Cell Biology and Genetics (44)

▢ Crash Course: Pathology (45)

▢ Crash Course: Pharmacology (46)

▢ Crush Step 1: The Ultimate USMLE Step 1 Review (47)

▢ Déjá Review: Biochemistry (48)

▢ Déjá Review: Neuroscience (49)

▢ Déjá Review: Pathology (50)

▢ Déjá Review: Pharmacology (51)

▢ Déjá Review: Physiology (52)

▢ Déjá Review: USMLE Step 1 (53)

▢ Digital Anatomist Project: Interactive Atlases (54)

▢ Doctors in Training COMLEX Level 1 Bundle (55)

▢ Doctors in Training Gross Anatomy & Radiology Study Guide (56)

▢ Doctors in Training OMM Review (57)

▢ Doctors in Training USMLE Step 1 Review Course (58)

▢ Dr. Najeeb Lectures (59)

▢ Elsevier's Integrated Immunology and Microbiology (60)

▢ Elsevier's Integrated Pharmacology (61)

▢ Elsevier's Integrated Physiology (62)

▢ Elsevier's Integrated Review: Genetics (63)

▢ Endocrine Physiology (64)

▢ Firecracker (65)

▢ First Aid Cases for the USMLE Step 1 (66)

▢ First Aid for the Basic Sciences: General Principles (67)

▢ First Aid for the Basic Sciences: Organ Systems (68)

▢ First Aid Q&A for the USMLE Step 1 (69)

▢ First Aid Step 1 Express (70)

▢ First Aid Step 1 Flash Facts (71)

▢ Goljan Rapid Review Pathology (72)

▢ Gray's Anatomy for Students Flash Cards (73)

▢ Hematology at a Glance (74)

▢ High-Yield Behavioral Science (75)

▢ High-Yield Biostatistics, Epidemiology, and Public Health (76)

▢ High-Yield Cell and Molecular Biology (77)

▢ High-Yield Embryology (78)

▢ High-Yield Gross Anatomy (79)

▢ High-Yield Histopathology (80)

▢ High-Yield Neuroanatomy (81)

▢ Jekel's Epidemiology, Biostatistics, Preventive Medicine, and Public Health (82)

▢ Kaplan Level 1 Qbank (83)

▢ Kaplan Medical Anatomy Flash Cards (84)

▢ Kaplan Medical USMLE Medical Ethics (85)

▢ Kaplan USMLE Step 1 Prep - In Center (86)

▢ Kaplan USMLE Step 1 Prep - Live (87)

▢ Kaplan USMLE Step 1 Prep - Live Online (88)

▢ Kaplan USMLE Step 1 Prep - On Demand (89)

▢ Kaplan USMLE Step 1 Qbank (90)

▢ Kaplan USMLE Step 1 Qbook (91)

▢ Katzung & Trevor's Pharmacology: Examination and Board Review (92)

▢ Lange Biochemistry and Genetics Flash Cards (93)

▢ Lange Microbiology & Infectious Diseases Flash Cards (94)

▢ Lange Pathology Flash Cards (95)

▢ Lange Pharmacology Flash Cards (96)

▢ Lippencott's Microcards: Microbiology Flash Cards (97)

▢ Lippincott's Illustrated Q&A Review of Rubin's Pathology (98)

▢ Lippincott's Illustrated Reviews: Biochemistry (99)

▢ Lippincott's Illustrated Reviews: Immunology (100)

▢ Lippincott's Illustrated Reviews: Microbiology (101)

▢ Lippincott's Illustrated Reviews: Pharmacology (102)

▢ Master the Boards USMLE Step 1 Pharmacology Flash Cards (103)

▢ medEssentials for the USMLE Step 1 (104)

▢ Medical Biochemistry - An Illustrated Review (105)

▢ Medical Microbiology and Immunology Flash Cards (106)

▢ Medical School Pathology (107)

▢ Memorang (108)

▢ Netter's Anatomy Flash Cards (109)

▢ Netter's Physiology Flash Cards (110)

▢ Northwestern Medical Review - Live Review Pack (111)

▢ Northwestern Medical Review - Online COMLEX Level 1 Review (112)

▢ Northwestern Medical Review - Online USMLE Step 1 Review (113)

▢ OMT Review: A Comprehensive Review in Osteopathic Medicine by Savarese (114)

▢ Osmosis (115)

▢ Packet Companion to Robbins and Cotran Pathologic Basis of Disease (116)

▢ Pass Program COMLEX-USA Live On-Site Program (117)

▢ Pass Program USMLE Step 1 Live Online Program (118)

▢ Pass Program USMLE Step 1 Live On-Site Program (119)

▢ Pathoma: Fundamentals of Pathology (120)

▢ Pathophysiology of Disease: Introduction to Clinical Medicine (121)

▢ PharmCards: Review Cards for Medical Students (122)

▢ Physeo (123)

▢ Picmonic (124)

▢ PreTest: Biochemistry and Genetics (125)

▢ PreTest: Clinical Vignettes for the USMLE Step 1 (126)

▢ Pretest: Microbiology (127)

▢ PreTest: Neuroscience (128)

▢ PreTest: Pathology (129)

▢ PreTest: Pharmacology (130)

▢ PreTest: Physiology (131)

▢ Pulmonary Pathophysiology: The Essentials (132)

▢ Radiopaedia (133)

▢ Rapid Review: Biochemistry (134)

▢ Rapid Review: Gross and Developmental Anatomy (135)

▢ Rapid Review: Microbiology and Immunology (136)

▢ Rapid Review: Pathology (137)

▢ Rapid Review: Pharmacology (138)

▢ Rapid Review: Physiology (139)

▢ Review of Medical Microbiology and Immunology (140)

▢ Review of Microbiology & Immunology (141)

▢ Robbins and Cotran Review of Pathology (142)

▢ SketchyMedical (143)

▢ Step-Up to USMLE Step 1 (144)

▢ The Pathology Guy (145)

▢ The Whole Brain Atlas (146)

▢ USMLE Consult (147)

▢ USMLE Images for the Boards: A Comprehensive Image-Based Review (148)

▢ USMLE Step 1 Made Ridiculously Simple (149)

▢ USMLE Step 1 Secrets in Color (150)

▢ USMLE Success Academy Live Step 1 Prep Program (151)

▢ USMLE Success Academy Online + Live Step 1 Prep Program (152)

▢ USMLE Success Academy Online Step 1 Prep Program (153)

▢ USMLEagle Prep - Step 1 (154)

▢ USMLE-Rx Step 1 Qmax (155)

▢ UWorld Qbank (156)

▢ Vander's Renal Physiology (157)

▢ WebPath: The Internet Pathology Laboratory (158)

▢ Wheater's Functional Histology: A Text and Colour Atlas (159)

▢ WolfPacc COMLEX Level 1 (160)

▢ WolfPacc USMLE Step 1 (161)

▢ Other (162) ________________________________________________

Q8 How many PRACTICE EXAMS did you purchase or plan to purchase in preparation for COMLEX-USA Level 1 / USMLE Step 1 Board Examinations?

NBME Step 1 (1)

o 0 (1)

o 1 (2)

o 2 (3)

o 3 (4)

o 4 (5)

o 5 (6)

o 6 (7)

NBOME Step 1 (2)

o 0 (1)

o 1 (2)

o 2 (3)

o 3 (4)

o (5)

o (6)

o (7)

Q9 Please select all resources you’ve purchased or plan to purchase in preparation for COMLEX-USA Level 2 CE / USMLE Step 2 CK Board Examinations

▢ N/A (1)

▢ A & L’s Review of Psychiatry (2)

▢ A & L’s Review of Surgery (3)

▢ Abernathy’s Surgical Secrets (4)

▢ Blueprints Clinical Cases in Family Medicine (5)

▢ Blueprints Clinical Cases in Medicine (6)

▢ Blueprints Clinical Cases in Neurology (7)

▢ Blueprints Clinical Cases in Obstetrics and Gynecology (8)

▢ Blueprints Clinical Cases in Pediatrics (9)

▢ Blueprints Clinical Cases in Psychiatry (10)

▢ Blueprints Clinical Cases in Surgery (11)

▢ Blueprints Medicine (12)

▢ Blueprints Neurology (13)

▢ Blueprints Obstetrics and Gynecology (14)

▢ Blueprints Pediatrics (15)

▢ Blueprints Psychiatry (16)

▢ Blueprints Q & A Step 2 Obstetrics & Gynecology (17)

▢ Blueprints Q & A Step 2 Pediatrics (18)

▢ Blueprints Q & A Step 2 Psychiatry (19)

▢ Blueprints Surgery (20)

▢ Boards & Wards for USMLE Step 2 (21)

▢ Case Files Emergency Medicine (22)

▢ Case Files Family Medicine (23)

▢ Case Files Internal Medicine (24)

▢ Case Files Obstetrics and Gynecology (25)

▢ Case Files Pediatrics (26)

▢ Case Files Psychiatry (27)

▢ Case Files Surgery (28)

▢ Clinical Vignettes for the USMLE Step 2 CK: PreTest Self-Assessment & Review (29)

▢ COMBANK Question Bank (30)

▢ COMQUEST Question Bank (31)

▢ Crush Step 2: The Ultimate USMLE Step 2 Review (32)

▢ Déjà Review: Emergency Medicine (33)

▢ Déjà Review: Family Medicine (34)

▢ Déjà Review: Internal Medicine (35)

▢ Déjà Review: Obstetrics and Gynecology (36)

▢ Déjà Review: Pediatrics (37)

▢ Déjà Review: Psychiatry (38)

▢ Déjà Review: Surgery (39)

▢ Déjà Review: USMLE Step 2 CK (40)

▢ Doctors in Training COMLEX Level 2 Bundle (41)

▢ Doctors in Training USMLE Step 2 CK Review Course (42)

▢ Dr. Pestana's Surgery Notes: Top 180 Vignettes for the Surgical Wards (43)

▢ Emergency Medicine: PreTest Self-Assessment and Review (44)

▢ Family Medicine: PreTest Self-Assessment and Review (45)

▢ First Aid Cases for the USMLE Step 2 CK (46)

▢ First Aid for the Emergency Medicine Clerkship (47)

▢ First Aid for the Medicine Clerkship (48)

▢ First Aid for the Obstetrics & Gynecology Clerkship (49)

▢ First Aid for the Pediatrics Clerkship (50)

▢ First Aid for the Psychiatry Clerkship (51)

▢ First Aid for the Surgery Clerkship (52)

▢ First Aid for the Wards (53)

▢ First Aid for USMLE Step 2 CK (54)

▢ First Aid Q&A for the USMLE Step 2 CK (55)

▢ High-Yield Internal Medicine (56)

▢ High-Yield Obstetrics and Gynecology (57)

▢ High-Yield Psychiatry (58)

▢ High-Yield Surgery (59)

▢ Images from the Wards: Diagnosis and Treatment (60)

▢ In A Page Emergency Medicine (61)

▢ In A Page Pediatrics (62)

▢ In A Page Surgery (63)

▢ Kaplan USMLE Step 2 CK Prep - In Center (64)

▢ Kaplan USMLE Step 2 CK Prep - Live (65)

▢ Kaplan USMLE Step 2 CK Prep - Live Online (66)

▢ Kaplan USMLE Step 2 CK Prep - On Demand (67)

▢ Kaplan USMLE Step 2 QBank (68)

▢ Lange Outline Review: USMLE Step 2 (69)

▢ Lange Practice Tests: USMLE Step 2 (70)

▢ Lange Q&A: Internal Medicine (71)

▢ Lange Q&A: Obstetrics and Gynecology (72)

▢ Lange Q&A: Pediatrics (73)

▢ Lange Q&A: Psychiatry (74)

▢ Lange Q&A: Surgery (75)

▢ Lange Q&A: USMLE Step 2 (76)

▢ Master the Boards USMLE Step 2 CK (77)

▢ Medicine: PreTest Self-Assessment & Review (78)

▢ Neurology Recall (79)

▢ Neurology Secrets (80)

▢ Neurology: PreTest Self-Assessment & Review (81)

▢ NMS Obstetrics and Gynecology (82)

▢ NMS Pediatrics (83)

▢ NMS Psychiatry (84)

▢ NMS Review for USMLE Step 2 CK (85)

▢ NMS Surgery (86)

▢ Northwestern Medical Review - Online COMLEX Level 2 CE Review (87)

▢ Northwestern Medical Review - Online USMLE Step 2CK Review (88)

▢ Obstetrics and Gynecology Recall (89)

▢ Obstetrics and Gynecology Secrets (90)

▢ Obstetrics and Gynecology: PreTest Assessment & Review (91)

▢ Pass Program USMLE Step 2 Live On-Demand Program (92)

▢ Pass Program USMLE Step 2 Live Online Program (93)

▢ Pass Program USMLE Step 2 Live On-Site Program (94)

▢ Pediatric Secrets (95)

▢ Pediatrics Recall (96)

▢ Pediatrics: PreTest Self-Assessment & Review (97)

▢ Physical Diagnosis: PreTest Self-Assessment & Revie (98)

▢ Psychiatry: PreTest Self-Assessment & Review (99)

▢ Review 2 Rounds: Visual Review and Clinical Reference (100)

▢ Step-Up to Medicine (101)

▢ Step-Up to USMLE Step 2 CK (102)

▢ Surgery: PreTest Self-Assessment & Review (103)

▢ Surgical Recall (104)

▢ Underground Clinical Vignettes Step 2: Emergency Medicine (105)

▢ Underground Clinical Vignettes Step 2: Internal Medicine Vols. I and II (106)

▢ Underground Clinical Vignettes Step 2: Neurology (107)

▢ Underground Clinical Vignettes Step 2: OB/GYN (108)

▢ Underground Clinical Vignettes Step 2: Pediatrics (109)

▢ Underground Clinical Vignettes Step 2: Psychiatry (110)

▢ Underground Clinical Vignettes Step 2: Surgery (111)

▢ Underground Clinical Vignettes: Step 2 Bundle (112)

▢ USMLE Consult’s Step 2 CK Question Bank (113)

▢ USMLE Road Map: Emergency Medicine (114)

▢ USMLE Step 2 Mock Exam (115)

▢ USMLE Step 2 Recall (116)

▢ USMLE Step 2 Secrets (117)

▢ USMLE Success Academy Live Step 2 CK Prep Program (118)

▢ USMLEagle Prep - Step 2 (119)

▢ USMLEasy (120)

▢ USMLE-Rx USMLE Step 2 Qmax (121)

▢ Uworld Step 2 CK Qbank (122)

▢ WolfPacc COMLEX Level 1 (123)

▢ WolfPacc USMLE Step 2 CK (124)

▢ Other (125) ________________________________________________

Q10 Please select all resources you’ve purchased or plan to purchase in preparation for COMLEX-USA Level 2 PE / USMLE Step 2 CS Board Examinations?

▢ N/A (1)

▢ Blueprints USMLE Step 2 CS (2)

▢ CS Checklists: Portable Review for the USMLE Step 2 CS (3)

▢ First Aid for the USMLE Step 2 CS (4)

▢ Mastering the USMLE Step 2 CS (5)

▢ NMS Review for the USMLE Clinical Skills Exam (6)

▢ Pass Program USMLE Step 2 Clinical Skills Program (7)

▢ The Ultimate Guide and Review for the USMLE Step 2 Clinical Skills Exam (8)

▢ USMLE Step 2 CS: Complex Cases-35 Cases You Are Likely to See on the Exam (9)

▢ Other (10) ________________________________________________

Q11 How many PRACTICE EXAMS did you purchase or plan to purchase in preparation for USMLE Step 2 CK / USMLE Step 2 CS / COMLEX-USA Level 2 CE?

NBME Step 2 CK (1)

o 0 (1)

o 1 (2)

o 2 (3)

o 3 (4)

NBME Step 2 CS (2)

o 0 (1)

o 1 (2)

o 2 (3)

o (4)

NBOME Level 2 CE (3)

o 0 (1)

o 1 (2)

o (3)

o (4)

Q12 Please evaluate the following: 

I plan to enter a Primary Care Residency (Family Medicine OR General Internal Medicine OR Pediatrics) (1)

o  Strongly Disagree (1)

o  Disagree (2)

o  Neutral (3)

o  Agree (4)

o  Strongly agree (5)

Preparation for Board Examinations influenced how I approached medical school. (2)

o  Strongly Disagree (1)

o  Disagree (2)

o  Neutral (3)

o  Agree (4)

o  Strongly agree (5)

I would advise future medical students to carefully plan for the cost of preparation for Board Examinations. (3)

o  Strongly Disagree (1)

o  Disagree (2)

o  Neutral (3)

o  Agree (4)

o  Strongly agree (5)

Studying for medical school courses took priority over studying for Board Examinations. (4)

o  Strongly Disagree (1)

o  Disagree (2)

o  Neutral (3)

o  Agree (4)

o  Strongly agree (5)

 My medical school's curriculum prepared me for Board Examinations. (5)

o  Strongly Disagree (1)

o  Disagree (2)

o  Neutral (3)

o  Agree (4)

o  Strongly agree (5)

My school purchased Board Examination preparation material that was integral for my preparation. (6)

o  Strongly Disagree (1)

o  Disagree (2)

o  Neutral (3)

o  Agree (4)

o  Strongly agree (5)
